# Changes in energy metabolism, and levels of stress-related hormones and electrolytes in horses after intravenous administration of romifidine and the peripheral α-2 adrenoceptor antagonist vatinoxan

**DOI:** 10.1186/s13028-018-0380-x

**Published:** 2018-05-09

**Authors:** Soile Anja Eliisa Pakkanen, Annemarie de Vries, Marja Riitta Raekallio, Anna Kristina Mykkänen, Mari Johanna Palviainen, Satu Marja Sankari, Outi Maritta Vainio

**Affiliations:** 1 Department of Equine and Small Animal Medicine, Faculty of Veterinary Medicine, University of Helsinki, P.O. Box 57, 00014 Helsinki, Finland; 2 Davies Veterinary Specialists, Manor Farm Business Park, Higham Gobion, Hertfordshire, UK

**Keywords:** α-2 adrenoceptor agonist, Glucose, Horse, Hyperglycaemia, Insulin, MK-467, Romifidine, Vatinoxan

## Abstract

**Background:**

Romifidine, an α-2 adrenoceptor agonist, is a widely-used sedative in equine medicine. Besides the desired sedative and analgesic actions, α-2 adrenoceptor agonists have side effects like alterations of plasma concentrations of glucose and certain stress-related hormones and metabolites in various species. Vatinoxan (previously known as MK-467), in turn, is an antagonist of α-2 adrenoceptors. Because vatinoxan does not cross the blood brain barrier in significant amounts, it has only minor effect on sedation induced by α-2 adrenoceptor agonists. Previously, vatinoxan is shown to prevent the hyperglycaemia, increase of plasma lactate concentration and the decrease of insulin and non-esterified free fatty acids (FFAs) caused by α-2 adrenoceptor agonists in different species. The aim of our study was to investigate the effects of intravenous romifidine and vatinoxan, alone and combined, on plasma concentrations of glucose and some stress-related hormones and metabolites in horses.

**Results:**

Plasma glucose concentration differed between all intravenous treatments: romifidine (80 μg/kg; ROM), vatinoxan (200 μg/kg; V) and the combination of these (ROM + V). Glucose concentration was the highest after ROM and the lowest after V. Serum FFA concentration was higher after V than after ROM or ROM + V. The baseline serum concentration of insulin varied widely between the individual horses. No differences were detected in serum insulin, cortisol or plasma adrenocorticotropic hormone (ACTH) concentrations between the treatments. Plasma lactate, serum triglyceride or blood sodium and chloride concentrations did not differ from baseline or between the treatments. Compared with baseline, plasma glucose concentration increased after ROM and ROM + V, serum cortisol, FFA and base excess increased after all treatments and plasma ACTH concentration increased after V. Serum insulin concentration decreased after V and blood potassium decreased after all treatments.

**Conclusions:**

Romifidine induced hyperglycaemia, which vatinoxan partially prevented despite of the variations in baseline levels of serum insulin. The effects of romifidine and vatinoxan on the insulin concentration in horses need further investigation.

## Background

Romifidine is an α-2 adrenoceptor agonist which is commonly used to induce sedation and analgesia in horses. In addition to their sedative and analgesic effects, α-2 adrenoceptor agonists have a marked effect on plasma concentrations of glucose and some stress-related hormones and metabolites; romifidine, xylazine and detomidine increase plasma glucose concentrations by inhibiting insulin release from pancreatic β-cells in horses [1, 2], ponies [3] and mice [4]. Detomidine is also reported to increase base excess (BE), indicating metabolic alkalosis [5] and to decrease the plasma concentration of free fatty acids (FFAs, or non-esterified free fatty acids) [3] and cortisol [3, 6] in horses. In contrast, in one study detomidine had no effect on plasma cortisol concentration [7]. Furthermore, plasma cortisol and adrenocorticotropic hormone (ACTH) concentrations decreased in horses after administration of clonidine, another α-2 adrenoceptor agonist [8]. The plasma concentration of potassium (K^+^) is reported to be unaffected by α-2 adrenoceptor agonists in horses [9]. In theory, decreased insulin concentration could result in hyperkalemia through diminished cellular K^+^ influx, as shown in dogs and humans [10].

The effects of α-2 adrenoceptor agonists on energy metabolism, stress-related hormones and metabolites can be attenuated by α-2 adrenoceptor antagonists. Atipamezole and yohimbine antagonized medetomidine-induced hyperglycaemia, hypoinsulinaemia and decrease in FFA concentration in dogs; the effect of atipamezole was reported to be dose-dependent [11]. Atipamezole also attenuated hyperglycaemia and decrease in FFA concentration in cats [12], and hyperglycaemia in cattle [13] and goats [14]. Contradictory results have also been reported; atipamezole did not affect hyperglycaemia [15] while the α-2 adrenoceptor antagonist tolazoline caused a rapid increase in plasma glucose concentration in horses treated with detomidine [3]. In addition, tolazoline attenuated the detomidine-induced decrease in FFA concentration transiently [3]. All of these α-2 adrenoceptor antagonists reverse, at least partly, both the sedative and analgesic effects induced by α-2 adrenoceptor agonist [3, 16–18], a feature which is often undesired.

Vatinoxan, previously known as MK-467 and L-659,066, is an α-2 adrenoceptor antagonist which poorly penetrates the blood brain barrier. Therefore, it targets mainly peripherally located α-2 adrenoceptors [19]. Vatinoxan prevented the hyperglycaemic effect of clonidine in humans [20] and mice [21]. In dogs, vatinoxan prevented the slight increase of plasma lactate and glucose concentrations and the decrease of insulin and FFA induced by the α-2 adrenoceptor agonist dexmedetomidine [22]. Vatinoxan, in addition, enhanced insulin responses to glucose in mice [21] and to exercise in humans [23]; although, in another study vatinoxan showed no effect on plasma glucose, insulin or insulin response to glucose in humans [24]. The effect of vatinoxan on quality of sedation induced by detomidine or romifidine in horses [25, 26] and dexmedetomidine in dogs [27–29] is only minor because it acts predominantly on peripheral α-2 adrenoceptors.

The aim of this study was to explore whether the intravenous (IV) administration of romifidine or vatinoxan induces changes in plasma concentrations of glucose or some metabolites and stress-related hormones in horses, and whether vatinoxan is able to antagonise the possible effects of romifidine when these agents are administered simultaneously. To the best of our knowledge, there are no previous reports of the effects of romifidine in combination with vatinoxan, on plasma concentrations of glucose or stress-related hormones and metabolites in horses or any other species.

## Methods

Seven Finnhorse mares aged 15 ± 5 years (mean ± SD) and with a body weight (BW) of 586 ± 44 kg were used in this study. The horses were considered healthy based on clinical examination (inspection of mucous membranes, measuring of capillary refill time and body temperature and auscultation of heart, lungs and intestinal sounds). Screening blood samples were not taken before the trials. The routine diet of these horses consisted of hay, silage and concentrates. On the day of the trials, the horses were fed normal hay and silage, but not concentrates, and had free access to water.

Baseline blood samples were drawn in the stables via puncture of jugular vein, after which the horses were taken to examination room and restrained in stocks. Two 14G, 80 mm intravenous catheters (Intraflon 2, Laboratoires Pharmaceutiques Vygon Uk Ltd., UK) were placed in the right jugular vein at least 15 cm apart under local infiltration of the skin with lidocaine (Lidocain 20 mg/mL, Orion Pharma, Espoo, Finland). An 18G, 70 cm central venous catheter (Cavafix Certo, B. Braun Melsungen AG, Melsungen, Germany) was placed in the left jugular vein and a 20G, 45 mm catheter (BD Arterial cannula, Becton–Dickinson India Pvt., India) was placed in the transverse facial artery under local skin infiltration with lidocaine. All catheters were sutured to the adjacent skin, except the caudal jugular catheter, which was used for the administration of medications and was immediately removed after injection. The remaining cranial jugular catheter was used for blood sampling. The arterial catheter was used for sampling for arterial blood gas analysis and for monitoring arterial blood pressure, and the central venous catheter for monitoring central venous blood pressure; these results are a subject of a separate paper [26]. The horses used in the study were research horses that were accustomed to minor procedures like IV punctures. Some of the horses mildly resisted their placement into the stocks and one horse got agitated by standing in the stocks for a long time without sedation. All the horses tolerated instrumentation without marked resistance. To obtain full immobility during arterial catheter placement, a twitch was used in all of the horses. All the catheters were removed at the end of the trial and replaced at the day of next trial.

Each horse was treated three times by means of a blinded cross-over Latin square design with a minimum washout period of 6 days. Venous blood samples were collected into EDTA, fluoride-oxalate and serum tubes in the stable (baseline) before each horse was taken to the examination room for instrumentation, administration of drugs and monitoring. After instrumentation, the horses were let to settle down for 5 min before administration of the medications. The horses received the following medications intravenously:

1. Romifidine hydrochloride (HCl) (80 μg/kg BW, Sedivet, Boehringer Ingelheim Vetmedica GmbH, Ingelheim, Germany; ROM). 2. Romifidine HCl (80 μg/kg BW) and Vatinoxan HCl (200 μg/kg BW, Vetcare Ltd, Salo, Finland; ROM + V). 3. Vatinoxan HCl (200 μg/kg BW; V).

Vatinoxan HCl was supplied as a powder. For each treatment, the drugs were diluted in saline (Natriumklorid, B. Braun Melsungen AG, Germany) in a single syringe to a total volume of 20 mL, and were administered IV as a bolus injection over 15 s at T0. Venous blood samples were taken 15, 30, 60, 90 and 120 min (T15–T120, respectively) after drug administration. EDTA and fluoride-oxalate tubes were placed in ice water immediately after blood sampling and centrifuged within 10 min to separate the plasma. Fluoride-oxalate plasma for analysis of lactate and glucose was refrigerated until analysis (maximum of 48 h). EDTA plasma was frozen at − 20 °C until ACTH analysis. Tubes for serum samples were kept at room temperature until centrifugation, which was performed on the same day. Serum was frozen at − 20 °C to await cortisol, FFA, triglyceride and insulin analyses. The laboratories analysing the blood samples were unaware of the treatments administered to the horses.

Lactate was analysed with enzymatic lactate oxidase (Konelab™ Lactate PAP, Thermo Fisher Scientific Ltd, Vantaa, Finland), glucose with the photometric glucose hexokinase 2-reagent method (Konelab™ Glucose HK) and triglycerides with the enzymatic colorimetric method (Konelab™ Trigycerides; Konelab 30i Clinical Chemistry Analyzer). The enzymatic colorimetric method was used for the determination of FFAs (NEFA-C, Wako Chemicals GmbH, Neuss, Germany; KONE Pro Selective Chemistry Analyzer, Thermo Fisher Scientific Ltd). ACTH was analysed with a solid-phase, two-site sequential chemiluminescent immunometric assay and insulin with a solid-phase, enzyme-labelled chemiluminescent immunometric assay (Immulite 2000, Siemens Healthcare Diagnostics Products GmbH, Marburg, Germany). Plasma cortisol concentration was analysed by RIA (Spectria cortisol RIA kit, Orion Diagnostica Ltd, Espoo, Finland), and samples were run as duplicates.

Venous blood gas samples (PICO50 blood gas syringes; Radiometer Medical ApS, Denmark) were taken after instrumentation (baseline) and at T5, T15, T30 and T60 after drug administration for measurement of potassium (K^+^), sodium (Na^+^), chloride (Cl^−^) and base excess (BE) (IDEXX VetSta; ME, USA).

The normality assumptions were evaluated with Shapiro–Wilk tests. Friedman’s two-way analysis of variance for related samples was used to compare serum insulin concentrations to baseline within each treatment. Kruskall–Wallis test was used for comparisons of serum insulin concentrations between the treatments. Concentrations of all other variables were compared between the treatments with repeated measures analysis of variance (rmANOVA) and pairwise comparisons within each time point were conducted with Student’s t test. All significance values were adjusted by Bonferroni correction for multiple tests. Significance was set at *P *< 0.05.

## Results

Romifidine induced sedation in all of the horses and vatinoxan did not significantly affect the quality of sedation. Vatinoxan administered alone caused mild abdominal discomfort and watery faeces in some of the horses, but otherwise it was well tolerated. When romifidine and vatinoxan were administered simultaneously, many of the peripheral cardiovascular and intestinal side-effects of romifidine were alleviated [26].

**Fig. 1:**
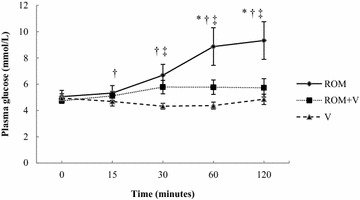
Mean plasma glucose concentration of seven horses. The horses received romifidine (ROM, 80 μg/kg IV), ROM with vatinoxan (ROM + V, 80 μg/kg + 200 μg/kg IV) and vatinoxan (V, 200 μg/kg IV) at T0. Error bars indicate the standard deviation. Plasma glucose concentrations were significantly different (*P *< 0.05) from baseline at T60 and T120 after ROM and at T30, T60 and T120 after ROM + V. Significant difference (*P *< 0.05) between * ROM and ROM + V, ^†^ROM + V and V and ^‡^ROM and V

Plasma glucose concentration showed significant differences between all treatments. The concentration was the highest after ROM and the lowest after V. Compared to baseline, ROM and ROM + V, but not V, significantly increased plasma glucose concentration (Fig. 1).

Serum insulin concentrations of five horses were within the laboratory reference range (0–19 µU/mL, Animal Laboratory Vetlab, Tampere, Finland) at all time-points. The remaining two horses had their values above the reference range at several measuring points; concentrations varied from below detection level (2 µU/mL)–82.9 µU/mL. No significant differences in insulin concentration were detected between treatments, but the inter-individual variation was high. Insulin concentration significantly decreased from baseline after V (Table 1).

**Table 1:** Serum insulin concentrations

	Baseline	T15	T30	T60	T120
ROM	6.0 (83)	< 2.0 (49)	< 2.0 (45)	< 2.0 (32)	< 2.0 (42)
ROM + V	4.8 (58)	< 2.0 (45)	< 2.0 (62)	< 2.0 (39)	< 2.0 (59)
V	7.8 (80)	3.2 (74)	< 2.0 (47)*	4.3 (58)	< 2.0 (43)*

Medians (and highest concentrations) of serum insulin concentration of seven horses (µIU/mL). The horses received romifidine (ROM, 80 μg/kg IV), ROM with vatinoxan (ROM + V, 80 μg/kg + 200 μg/kg IV) and MK-467 (V, 200 μg/kg IV) at T0. The lowest concentration after all treatments at all time points was below the detection limit (< 2 µIU/mL)

* Significantly different (*P *< 0.05) from baseline (T0), no significant differences detected between treatments

**Fig. 2:**
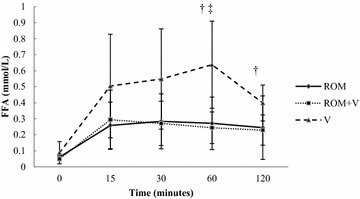
Mean serum FFA concentration of seven horses. The horses received romifidine (ROM, 80 μg/kg IV), ROM with vatinoxan (ROM + V, 80 μg/kg + 200 μg/kg IV) and vatinoxan (V, 200 μg/kg IV) at T0. Error bars indicate the standard deviation. Serum FFA concentrations were significantly different (*P *< 0.05) from baseline at T30 after ROM, at T15 and T30 after ROM + V and at T30 and T60 after V. Significant difference (*P *< 0.05) between ^†^ROM + V and V and ^‡^ROM and V

Serum FFA concentration was higher after V than ROM or ROM + V. Serum FFA concentrations increased from baseline after all treatments (Fig. 2).

Baseline plasma ACTH concentrations were within the reference range (< 35 pg/mL, Animal Laboratory Vetlab) in all but one horse, which had a higher concentration before ROM + V. After the treatments, the values varied from 5.82 to 74.9 pg/mL, except for one measurement in one horse of 185 pg/mL at T120 after ROM. This reading was confirmed by measuring in duplicate. Plasma ACTH concentrations, relative to baseline, increased after MK at T15 and T30, but significant differences were not detected between treatments. Plasma lactate and serum triglyceride concentrations did not differ significantly from baseline or between treatments. Serum cortisol concentrations increased from baseline after all treatments, but no significant differences emerged between treatments (Table 2).

**Table 2:** Plasma ACTH and lactate, serum triglyceride, cortisol and blood K^+^, Na^+^ and BE concentrations

Analyte	Treatment	Baseline	T15	T30	T60	T120
ACTH	ROM	16.8 (± 3.83)	32.3 (± 22.4)	25.0 (± 12.5)	18.7 (± 7.66)	44.7 (± 62.4)
	ROM + V	19.4 (± 11.1)	28.5 (± 12.4)	21.6 (± 8.39)	21.4 (± 13.2)	19.1 (± 11.1)
	V	17.3 (± 6.53)	32.2* (± 6.66)	29.0* (± 5.71)	23.0 (± 5.68)	16.1 (± 5.68)
Lactate	ROM	0.60 (± 0.30)	0.61 (± 0.17)	0.63 (± 0.24)	0.49 (± 0.11)	0.41 (± 0.24)
	ROM + V	0.58 (± 0.29)	0.69 (± 0.24)	0.70 (± 0.27)	0.71 (± 0.24)	0.56 (± 0.17)
	V	0.50 (± 0.59)	0.55 (± 0.35)	0.55 (± 0.33)	0.61 (± 0.30)	0.70 (± 0.35)
Triglycerides	ROM	0.30 (± 0.10)	0.36 (± 0.13)	0.37 (± 0.13)	0.36 (± 0.13)	0.27 (± 0.07)
	ROM + V	0.27 (± 0.03)	0.30 (± 0.06)	0.30 (± 0.09)	0.29 (± 0.12)	0.24 (± 0.12)
	V	0.28 (± 0.08)	0.32 (± 0.09)	0.33 (± 0.11)	0.30 (± 0.10)	0.23 (± 0.11)
Cortisol	ROM	59.0 (± 13.9)	120* (± 34.7)	106 (± 37.6)	86.6 (± 35.4)	113 (± 51.7)
	ROM + V	51.9 (± 16.1)	107* (± 24.7)	84.1 (± 23.1)	76.5 (± 29.0)	66.3 (± 26.5)
	V	61.2 (± 14.7)	131* (± 34.2)	120* (± 30.4)	102 (± 23.5)	79.4 (± 32.7)

Analyte	Treatment	Baseline	T5	T15	T30	T60
BE	ROM	4.00 (± 0.54)	4.04 (± 0.95)	4.47 (± 0.47)	4.76 (± 0.79)	5.74* (± 0.88)
	ROM + V	4.46 (± 1.43)	4.19 (± 1.32)	4.31 (± 1.55)	4.84 (± 1.30)	5.41 (± 1.06)
	V	3.86 (± 0.78)	3.63 (± 0.87)	3.96 (± 0.96)	4.19 (± 0.66)	5.24* (± 0.57)
K^+^	ROM	4.30 (± 0.26)	4.16 (± 0.28)	4.09* (± 0.28)	4.16 (± 0.30)	4.17 (± 0.27)
	ROM + V	4.20 (± 0.29)	4.11 (± 0.40)	3.87*^,$^ (± 0.28)	3.91 (± 0.25)	3.84* (± 0.27)
	V	4.44 (± 0.36)	4.39 (± 0.28)	4.30^$^ (± 0.31)	4.21 (± 0.36)	3.94* (± 0.31)

Means (± SD) of plasma ACTH (pg/mL), plasma lactate (mmol/L), serum triglyceride (mmol/L), serum cortisol (nmol/mL) and venous blood K^+^and BE (mmol/L) concentrations of seven horses. The horses received romifidine (ROM, 80 μg/kg IV), ROM with vatinoxan (ROM + V, 80 μg/kg + 200 μg/kg IV) and vatinoxan (V, 200 μg/kg IV) at T0

* Significantly different (*P *< 0.05) from baseline (T0)

^$^ Significant difference between treatments

Base excess showed an increasing trend after all treatments and there was a significant difference between baseline and T60 after ROM and V, but no significant differences were detected between the treatments. Potassium, in contrast, decreased after all the treatments and the concentrations were significantly lower than baseline at T15 after ROM and ROM + V, and at T60 after ROM + V and V. There was also a significant difference in K^+^ concentrations between ROM + V and V at T15 (Table 2). Sodium and Cl^−^ concentration were stable trough the monitoring period (data not shown).

## Discussion

As far as we are aware, this is the first study reporting the effects of romifidine administered in combination with vatinoxan, on plasma concentrations of glucose or hormones and metabolites in any animal species. In this study, romifidine induced hyperglycaemia in horses and the plasma glucose concentration remained high until the end of the monitoring period of 120 min. This indicates that the effect of romifidine on plasma glucose concentration is long lasting and that plasma glucose concentration may, indeed, have peaked after the last time point of this study. These findings are in agreement with previous studies from horses treated with romifidine [1], detomidine [3, 18] and dogs treated with xylazine, medetomidine and dexmedetomidine [22, 30]. In contrast, Raekallio and co-workers [31] reported a decrease in plasma glucose concentration in dogs after dexmedetomidine administration, but they noted that their monitoring period of 90 min might have been too short to detect possible hyperglycaemia. It is noticeable that the sedative effect of romifidine was already ceased at the point of highest plasma glucose concentration [26], which means that its effect on energy metabolism is longer lasting than the sedative action.

Plasma glucose concentration also increased after ROM + V, although to a lesser extent than after ROM. This suggests that V partially prevented romifidine-induced hyperglycaemia. Prevention of the haemodynamic effects of dexmedetomidine by vatinoxan in dogs is reported to be dose-dependent [32]. This could also apply to the effects of vatinoxan on alterations in plasma hormone and metabolite concentrations caused by alpha-2 adrenoceptor agonists. On that basis, we assumed that the dose of 200 µg/kg used in our study might have been inadequate for complete prevention of romifidine-induced hyperglycaemia. In dogs, for example, vatinoxan completely antagonized dexmedetomidine (10 µg/kg) induced hyperglycaemia at the dose of 500 µg/kg [22]. Of the other α-2 adrenoceptor antagonists, antagonism of medetomidine induced hyperglycaemia and hypoinsulinaemia in dogs by atipamezole is also reported to be dose-dependent [11]. In humans, vatinoxan partially inhibited both the clonidine-induced increase of plasma glucose and reduction of plasma insulin concentration. These authors speculated that glucose homeostasis could be only partially regulated by peripheral α-2 adrenoceptors, which would also explain the incompleteness of inhibition [33].

In our study, vatinoxan, when administered alone, did not significantly affect plasma glucose concentration. This finding is in agreement with previous reports in which vatinoxan did not change plasma glucose or insulin concentrations in normoglycaemic fasted mice [21] or humans [33, 34]. In contrast, vatinoxan increased insulin and decreased plasma glucose concentration in hyperglycaemic mice [21]. However, despite no significant difference in plasma glucose concentration, vatinoxan decreased serum insulin concentration in the present study. This might relate to the balance between inhibition of α-2 adrenoceptors in pancreatic β-cells and increased sympathoadrenal output and concentration of catecholamines due to stress caused by restraint of non-sedated horses in stocks. The ability of vatinoxan to decrease serum insulin in horses could be a useful feature in horses suffering from equine metabolic syndrome (EMS), as hyperinsulinaemia is known to predispose these horses to laminitis [35].

Alpha-2 adrenoceptor agonist-related hyperglycaemia is known to result from a reduction of insulin release by a direct action of the α-2 adrenoceptor agonist on pancreatic β-cells [4]. However, in our study this association between hyperglycaemia and hypoinsulinaemia could not be demonstrated, because in the majority of the samples the serum insulin concentration was below the detection limit of the analysing method. In individual horses, romifidine seemed to cause an increase in plasma glucose concentration, while the insulin concentration stayed below the detection limit. This could suggest that even a slight decrease of insulin concentration, which was not detectable in the analysis, may result in hyperglycaemia or there could have been other factors affecting the increase of glucose concentration. Findings in previous studies [11, 30, 36] suggest that the α-2-mediated decrease in plasma insulin concentration might not be the only factor affecting blood glucose concentration in animals treated with α-2 adrenoceptor agonists. We also suggest that some of the effects of romifidine on glucose concentration might have been other than α-2 adrenoceptor activation in pancreas, such as hepatic glycogenolysis induced by α-2-adrenergic agents and gluconeogenesis mediated by α-1 adrenoceptors, as romifidine is stated to be partial α-2 adrenoceptor agonist [37] or to have α-2:α-1 selectivity of 340:1 [38]. Some of the effects might also be mediated by central imidazoline receptors, as romifidine is an imidazoline derivative.

Serum cortisol concentration increased rather quickly after each treatment, after which it slowly decreased again. Plasma ACTH concentrations showed a similar trend, although the increase was significant only after V. These increases are probably due to stress induced by instrumentation and confinement to stocks. Instrumentation procedure was the same before every trial and we assume that the bias caused by stress from instrumentation would be similar throughout the experiment. Short term increases in serum cortisol concentration could be of clinical importance in EMS or pituitary pars intermedia dysfunction (PPID) patients because they are reported to enhance vasoconstriction in laminar veins in vitro, creating a possible risk factor for laminitis [39]. There are conflicting reports on the effects of α-2 adrenoceptor agonists on cortisol concentration in various species. Plasma cortisol concentration is reported to either decrease or to remain constant in horses [3, 6, 7], to remain constant in various other species such as dogs [11, 22, 30] and calves [13] or to increase in dairy cows, sheep and goats [13, 14]. Other factors, such as differences between species, differences in baseline cortisol concentrations and stress related to the environment or sampling procedures, likely affected cortisol concentrations in these studies. Cortisol concentration is known to fluctuate during the day in horses; it is highest from morning until early afternoon (8 a.m.–14 p.m.) and lowest before midnight, around 10–12 p.m. [40–42]. To avoid the effect of diurnal variation in our results, all three treatments were conducted at approximately the same time of the day for each horse.

In the present study, we detected an increase in serum concentrations of FFA after each treatment. The concentration of FFA was significantly higher after V than after ROM or ROM + V, which, in turn, did not differ significantly from each other. This was indicative of stress caused by instrumentation, which most likely increased catecholamine plasma concentrations. This was manifested as increased FFA concentration. Alpha-2 adrenoceptor agonists are previously reported to decrease serum FFA in horses [3] and dogs [22, 30]. In our study, romifidine-induced sedation probably affected serum FFA concentration only partially because the stress increasing the FFA concentrations was induced before the horses received the medications. The stress caused by procedures may also overcome the decreasing actions of α-2 adrenoceptor agonists on serum FFA, which is reported in horses undergoing standing laparoscopy [43]. The greater increase in serum FFA after V could also result partly from a direct effect of vatinoxan on lipolysis as vatinoxan is reported to increase the plasma concentration of FFA [23] and, furthermore, α-2 adrenoceptor antagonists potentiate the lipolytic action of adrenaline in humans [44]. In dogs, plasma FFA concentration showed a biphasic response after the combined administration of vatinoxan with dexmedetomidine. Initially, a similar decrease was noticed after both medications, but later the concentration increased significantly more after the combined administration [22]. The temporal discrepancy was speculated to result from the balance between catecholamine and insulin concentrations since both affect the activity of hormone-sensitive lipase, which is the main regulator of lipolysis [45].

As all lactate concentrations were within the reference range (< 1.5 mmol/L, Laboratory Ellab, Ypäjä, Finland) and the concentrations did not differ significantly from baseline values, which indicates that there was no evidence of tissue hypoxia or lactic acidosis after any of the treatments. In fact, as the BE increased after all treatments, the metabolic state of the horses changed towards metabolic alkalosis during the experiment. This observation is in agreement with previous findings of Raekallio and co-workers [5]. Possible reasons for the change toward alkalosis after romifidine administration in the present study are altered renal function and compensatory response to respiratory acidosis due to decreased respiratory rate and increased arterial carbon dioxide tension [26]. As there was significant increase in BE during this relatively short sampling period, further investigation is needed to examine if the metabolic alkalosis will be clinically relevant for example after long infusions of α-2 adrenoceptor agonists for veterinary standing procedures that have become more common in recent years.

The blood K^+^ concentrations of the horses in our study decreased although the change was not clinically relevant. The regulation of blood potassium concentration is complex and involves, for example, aldosterone, insulin, sympathomimetics, and is regulated by the acid–base balance and renal function. Because of the high concentration difference between intracellular and extracellular potassium content, alterations in blood potassium concentrations are often a result of redistribution of potassium between these compartments [46]. Renal excretion of potassium is increased during metabolic alkalosis. Furthermore, both urine production and renal excretion of electrolytes are increased by alpha-2 adrenoceptor agonists [9], and one possible explanation of decreased potassium in the present study is increased loss to urine. Considering the complexity of potassium homeostasis, the effects of romifidine and vatinoxan on blood potassium need further investigation.

## Conclusions

Romifidine induced hyperglycaemia in horses is similar to other α-2 adrenoceptor agonists, but its effects on serum insulin warrant further investigations. Vatinoxan was able to alleviate the romifidine induced hyperglycaemia despite of the wide variation in baseline insulin concentrations. Further research is needed to evaluate the metabolic changes under stressful situations, such as veterinary procedures or in compromised patients (e.g. critically ill foals and adult horses with underlying disease processes such as PPID and EMS). In these patients, the metabolic and hormonal changes induced by α-2 adrenoceptor agonists may be of clinical importance, especially because they last longer than the sedative action of romifidine.
